# Knowledge and Associated Factors about Rare Diseases among Dentists in Israel: A Cross Sectional Survey

**DOI:** 10.3390/ijerph18136830

**Published:** 2021-06-25

**Authors:** Eitan Mijiritsky, Michal Dekel-Steinkeller, Oren Peleg, Shlomi Kleinman, Clariel Ianculovici, Amir Shuster, Shimrit Arbel, Menachem Ben-Ezra, Maayan Shacham

**Affiliations:** 1 The Maurice and Gabriela Goldschleger School of Dental Medicine, Tel Aviv University, Tel Aviv 6997801, Israel; michal@taldekel.com; 2 Department of Otolaryngology, Head and Neck and Maxillofacial Surgery, Tel-Aviv Sourasky Medical Center, Sackler Faculty of Medicine, Tel Aviv 6139001, Israel; orenpeleg@gmail.com (O.P.); dr.shlomi@gmail.com (S.K.); dr.clariel@gmail.com (C.I.); shusterim@gmail.com (A.S.); shimritarbel@gmail.com (S.A.); 3 School of Social Work, Ariel University, Ariel 40700, Israel; menbe@ariel.ac.il (M.B.-E.); Drmaayanshacham@gmail.com (M.S.)

**Keywords:** rare diseases, rare diseases with orofacial manifestation, dentists, knowledge, factors associated with knowledge, oral medicine

## Abstract

*Aim:* The current study aims to assess levels of knowledge among Israeli dentists about rare diseases with orofacial manifestations, and whether occupational, regional and social factors influence those levels. *Materials and Methods:* A total of 309 Israeli dentists participated in an online survey that provided basic demographic information pertaining to their knowledge about rare diseases, their clinical experience with rare diseases, what further information they considered necessary, and which sources of information they most frequently utilize. *Results:* Young age, country of dental education, practicing in both public and private settings, as well as the number of hours allocated to dental studies and the opportunity to acquire information on rare diseases with orofacial manifestations, all seem to affect the level of knowledge. *Conclusions:* Developments in the field of rare disease are constantly ongoing, and improvements in post-graduate dental studies about them should keep pace. The results of the current study reveal the areas upon which such curricula should focus with respect to dental practitioners.

## 1. Introduction

Dentists may be instrumental in the crucial and timely diagnosis of rare diseases with orofacial manifestations which may be among the first signs of their presence. Knowledge and resultant recognition of such manifestations are even more important when considering that some of those rare diseases may have already undergone irreversible damage before their orofacial manifestations [1]. Some of the rare diseases that include orofacial expressions are uncommon, and 471 of them were diagnosed as of 2017 [2], including those with particular significance to certain dental specialties, such as orthodontics [3] and periodontics [4]. The knowledge of rare diseases with orofacial manifestations is nevertheless relevant to all dental practitioners who may be the first to recognize the underlying pathology. 

Some diseases may be rare in certain areas of the world and more common in others, making the definition of “rare” subject to regional considerations. For example, the definition of a rare disease in the United States is one that affects fewer than 200,000 people across the 50 states [5,6], while a disease is considered rare by the European Union (EU) if it affects fewer than 5 out of 10,000 people across the EU [6,7]. There are about 6000–8000 rare diseases known to date, with the numbers increasing each day [6,8]. About 15% of such diseases may present with orofacial manifestation [5,7]. The establishment a diagnosis of a rare disease is known to suffer from delay, estimated at 4.8 years on average [9,10]. A recently published debate [8] proposed the notion that practitioners should follow their own gut feelings, in addition to the utilization of standard diagnostic tools, in order to arrive at a diagnosis more quickly. In their review, Svenstrup et al. [11] offered ways to aid in the search for information on rare diseases, such as specialized search engines and data mining tools. 

In their recent systematic review on evidence-based clinical practice for rare diseases, Rath et al. [12] listed several barriers to correct identification of those pathologies, among them rarity and limited knowledge. Studies on the knowledge, attitudes, and practices (KAPs) of general practitioners [13], and of dental practitioners [7], are sparse with regard to rare diseases, unlike the plethora of KAP studies on more common diseases, e.g., COVID-19 [14,15,16]. If rare diseases go undetected, it might result in high mortality rates [9,10]. This emphasizes the need to enhance and broaden dentists’ levels of knowledge about oral diseases, including rare diseases with orofacial manifestation. Dentists worldwide, Israeli dentists included, can benefit from training about this topic in order to prevent preventable mortality rates. 

The current study sought to assess the levels of knowledge and associated factors of rare diseases with orofacial manifestations among Israeli dentists. We employed the model described by Kühne et al. [7], and sought to assess whether occupational, regional and social factors influence the participants’ influence levels of knowledge. Given that a disease considered rare in one part of the world may be less rare and even commonplace in another, we sought to assess which rare diseases with orofacial manifestations are recognized by Israeli dentists and the factors that might influence the levels of such recognition.

## 2. Materials and Methods

### 2.1. Sampling and Procedure

Ethical approval for the current study was granted by the Institutional Review Board of the authors’ universities (E.M., M.B.-E., M.S.). An online platform (www.imkforms.com, accessed on 8 January 2021) was used to conduct the survey. Potential participants were approached via social media (WhatsApp, Facebook), along with personal contacts and subsequent snowball sampling, all of which included a description of the study and its objectives, along with an assurance of anonymity. Each participant then signed an electronic informed consent form. Data were collected from 309 Israeli dentists from 8 January 2021 to 7 February 2021.

### 2.2. Measurements

The current survey items were adapted based on a previous study [7,13]. They were back-translated from English to Hebrew by the authors (E.M., M.S.). The following basic demographics were collected: age (in years), sex (coded as “1 = male”, “2 = female”), dental experience (in years), dental specialty (coded as “1 = General dental practitioners” (GDP), “2 = Specialists in oral medicine or oral and maxillofacial surgery” (OMF), “3 = Specialists in Orthodontics, Periodontics, Pediatrics, Endodontics” (REST), “4 = Specialists in Prosthodontics” (PROS), country of study and degree acquisition (coded as “1 = Israel”, “2 = rest of world”), and work setting (coded as “1 = public”, “2 = private”, 3 = “military”, 4 = “public and private”). Both dental specialties and country of graduation variables were grouped into different groups based on field of expertise and distribution of participants in the current study. Prosthodontists received an individual coding due to having a large number of participants compared to other dental specialists.

In addition, the following self-reported measures were used (Supplementary Table S1 briefly describes the following):

### 2.3. Familiarization with Rare Diseases Presenting with Craniomaxillofacial Expression

The following items were used to estimate participants’ definitions of rare diseases with craniomaxillofacial expression: 

“How do you assess your level of knowledge about rare diseases?” (coded as “1 = inadequate”, “2 = nearly adequate”, “3 = adequate”, “4 = nearly good”, “5 = good”, “6 = very good”, “7 = excellent”).

“In your opinion, a rare disease is…?” (single choice, coded as “1 = I don’t know how to define a rare disease”, “2 = a life-threatening chronic disease, which is often genetically determined and difficult to cure”, “3 = a disease of which no more than 5 of 10,000 people in the EU are affected” (CORRECT ANSWER), “4 = a disease of which no more than 5 of 250,000 people in the EU are affected”).

“What percent of rare diseases manifest themselves in the craniomaxillofacial region?” (single choice, coded as “1 = 5%”, “2 = 7.5%”, “3 = 12%”, “4 = 15%” (CORRECT ANSWER), “5 = 32%”).

“Please estimate how long it takes for a rare disease with orofacial manifestation to be diagnosed as such after the first appearance of symptoms.” (single choice, coded as “1 = within the first month”, “2 = between 1 to 6 months”, “3 = between 6 to 18 months”, “4 = between 1.5 to 3 years”, “5 = after more than 3 years” (CORRECT ANSWER), “6 = no estimation”).

“Do you believe that your knowledge of rare diseases is sufficient?” (single choice, coded as “1 = not at all”, “2 = slightly sufficient”, “3 = moderately sufficient”, “4 = very sufficient”, “5 = extremely sufficient”).

### 2.4. Specific Rare Diseases with Orofacial Expression

Participants were asked to check whether they are familiar with certain rare disease which can manifest in the oromaxillofacial region. The disease list included Down syndrome, Ehlers–Danlos syndrome, Ectodermal dysplasia, Epidermolysis bullosa, Fetal alcohol syndrome, Gorlin–Goltz syndrome, Behçet’s disease, Crohn’s disease, Osteogenesis imperfecta, Bullous pemphigoid, Pemphigus vulgaris, Scleroderma, Von Willebrand–Jürgens syndrome, and X-linked-Hypophosphatemia. These diseases were included in the current study based on the study conducted by Kühne et al. [7]. An outcome variable, “Total rare diseases index” was created based upon the number of rare diseases each participant marked as being familiar with. Scoring ranged from 0 to 14.

### 2.5. Experience with Rare Diseases

The following items were used to estimate each participant’s experience with rare diseases: 

“Have you ever treated a patient affected by a rare disease/have you ever seen such a patient before?” (coded as “1 = no”, “2 = yes”).

“While performing a clinical and radiographic examination, do you think about a possible diagnosis of a rare disease as part of the anamnesis procedure?” (coded as “1 = no”, “2 = yes”).

“Have you ever diagnosed a rare disease with orofacial manifestations?” (coded as “1 = no”, “2 = yes, once”, “3 = yes, several times”).

“Was any time spent on acquiring knowledge about rare diseases with orofacial manifestations, including their diagnosis and therapy, during your dental education?” (coded as “1 = no”, “2 = no, that is why I have a lack of knowledge about rare diseases with orofacial manifestations”, “3 = yes”, “4 = yes, but too little time was spent on acquiring information about them”, “5 = yes, sufficient time was spent on acquiring information about them”).

“Have you attended training courses with focus on rare diseases with orofacial manifestations?” (coded as “1 = no, I am not interested in training courses with focus on rare diseases”, “2 = no, but I would like to attend such courses”, “3 = yes”).

“Do you know where to get information about diagnostics, course of disease and therapy when treating a patient affected by a rare disease with orofacial manifestations?” (coded as “1 = no”, “2 = yes”).

### 2.6. Sources of Knowledge about Rare Diseases

The following items were used to evaluate which sources of information participants’ turn to when in need of information and further education regarding rare diseases. 

*“Do you need information on rare diseases with orofacial manifestations in your everyday dental practice?”* (multiple answers, coded as “1 = no, because I am not interested in such information”, “2 = no, I am sufficiently informed”, “3 = yes, but, unfortunately, I do not have time for research”, “4 = yes, but I do not know where to get this information”, “5 = yes”).

*“Which of the following sources do you use for information about rare diseases?”* (multiple answers, coded as “1 = social media (Facebook, YouTube, etc.)”, “2 = dental friends/colleagues”, “3 = knowledge I acquired during my dental studies (excluding dental specialization, if applicable)”, “4 = knowledge I acquired during my dental studies (including dental specialization, if applicable)”, “5 = journals”, “6 = dedicated dental forums”).

“Which of the following organizations, websites and sources of information about rare diseases with orofacial manifestations do you know?” (multiple answers, coded as “1 = Israeli oral medicine association”, “2 = Israeli dental association”, “3 = other dental specialty association in Israel (pedodontics, periodontics, orthodontics, prosthodontics, oral and maxillofacial surgery)”, “4 = non-Israeli dental associations”, “5 = none of the above”).

In addition, two items were asked to evaluate the participants’ grasp of the importance of rare diseases with orofacial expression, and how their knowledge may be further expanded.

“I need information about rare diseases with orofacial manifestations in terms of…” (multiple answers, coded as “1 = incidence and prevalence”, “2 = lethality and mortality”, “3 = treatment options”, “4 = relevant medications”).

*“Do you, as a dentist, consider it important to have knowledge about rare diseases with orofacial manifestation?”* (multiple answers, coded as “1 = no, rare diseases play virtually no role in everyday dental practice”, 2 = “no, it is not important”, “3 = one should have heard about rare diseases”, “4 = yes, knowledge about rare diseases has an important differential diagnostic significance”, “5 = yes, it is a very important field”).

### 2.7. Statistical Analyses

Descriptive data were used to describe the characteristics of the sample, both in terms of demographics and the scores of different items. Group comparisons were carried out by analyses of variance (ANOVA) with the *Scheffé’s* test used for post hoc comparisons. Significance was set at α ≤ 0.05. 

In addition, a linear regression analysis was conducted utilizing the outcome variable “Total rare diseases” as a dependent variable, with the following serving as independent variables: age, sex, country of dental education and degree acquisition (Israel/other countries), work setting, the items “How do you assess your level of knowledge about rare diseases?”, “Do you consider your knowledge about rare disease as being sufficient?”, and “experience with rare diseases” items (sub-Section 2.5). Analyses were conducted with the SPSS version 25 (IBM, Armonk, NY, USA).

## 3. Results

A total of 309 responders participated in the current study. The variables dental specialties and country of graduation were grouped according to field of expertise and distribution of countries in the current study. Table 1 and Table 2 depict the distribution of the demographics of age, sex, years of dental experience, dental specialties, country of dental education and degree acquisition, and dental work setting.

Most of participants were males (*n* = 192, 62.1%), general dental practitioners (*n* = 211, 68.3%), received their dental education and certification in Israel (*n* = 225, 72.8%), and work in private (*n* = 152, 49.2%) and both private and public dental settings (n = 109, 35.2%) (Table 1). We therefore included the latter as a single variable (*n* = 84, 27.2%) (Table 2). Since oral medicine specialists and oral and maxillofacial surgeons in Israel spend vast amounts of time in studying oral pathology and oral medicine, they were assigned to one group (OMF). It emerged that the number of specialists in oral rehabilitation (prosthodontics) who participated in our study was equivalent to the combined specialists in dental specialties other than the OMF group, and were assigned to a group of their own (PROS). This reflects the 2016 statistic that most dental specialists in Israel are prosthodontists according to the Israeli Ministry of Health [17].

Table 3 depicts the distribution of answers regarding rare disease definition, prevalence, and time until such a diagnosis is obtained. The majority of dentists in all groups correctly answered the item on the definition of a rare disease (Table 3, range 36.4–57.1%). Most of the dentists across the different groups answered that 5% of rare diseases will present in the oromaxillofacial region (Table 3, range 33.3–43.6%). Between 10 and 19% of the dentists correctly answered the item on how long it takes to establish a definitive diagnosis about a rare disease: the majority of dentists (incorrectly) estimated a range of up to 3 years, while few answered correctly (more than 3 years; Table 3, 3–6.8%).

A regression analysis was used to test whether the independent variables used in the analysis significantly predicted the participants’ knowledge and familiarization of rare diseases with oromaxillofacial manifestations (Table 4). The results indicated that the predictors that had been used explained 31.5% of the variance (R2 = 0.342, F (12,295) = 12.76, *p* < 0.001). It should be noted that no collinearity issues were found, except for age with years of experience, and so years of experience was omitted from the regression analysis for that reason. It emerged that age (β = −0.253, *p* < 0.001), country of study and degree acquisition (β = −0.358, *p* < 0.001), dental work setting (β = 0.107, *p* < 0.05), enough hours having been allocated during studies (β = 0.111, *p* < 0.05), and knowing where to look for information about rare diseases (β = 0.117, *p* < 0.05) significantly predicted the familiarization of dentists with the number of rare diseases with oromaxillofacial presentation. In addition, Table 5 provides the percentage of dentists who previously either diagnosed or treated a patient presenting with a rare disease with oromaxillofacial manifestation. As noted, most dentists had treated such patients (range 70.1–95.2%), with the OMF group demonstrating higher numbers for encountering and for previously diagnosing those cases. Table 6 demonstrates the distribution of dentists who recognize and are familiar with various rare diseases with oromaxillofacial components.

Post hoc comparisons by means of *Scheffé’s* test were used to assess differences within items in the domain of sources of knowledge about rare diseases (Table 7. Those comparisons yielded significant differences between the two items “*Do you need information on rare diseases with orofacial manifestations in your everyday dental practice?*” and “*Which of the following sources do you use for information about rare diseases?*”. There were no statistically significant differences between the rest of the items included in that domain. The GDP participants reported that they require further information on rare diseases with oromaxillofacial presentation as part of their daily dental routine (39.3% of GDP), which was significantly different (*p* < 0.05) compared to the OMF group in which only one participant responded similarly (Table 7). Most of the dentists responded that they acquired their knowledge on such diseases as part of their dental studies (39.4–77.3%) (Table 7). One exception was the group of specialists in prosthodontics whose difference from other groups was significant (REST, *p* < 0.001, OMF, *p* < 0.01): only 39.4% of them stated they had acquired their knowledge about rare diseases during their specialization studies, as opposed to 76.2% of the OMF and 77.3% of the REST groups. High numbers of dentists turn to related journals when in need of information regarding rare diseases (range 50.2–69.7%). Dentists also indicated that dental colleagues were a viable and frequent source of information (range 47.6–75.8%).

Most of the dentist responded that they are familiar with and turn to the Israeli Oral Medicine Association as a source of information about rare diseases (56.8–75.8% of participants) (Table 7), and most believe that they need to expand their knowledge on different treatment modalities (77.3–90.5%). Finally, most dentists (66.7–81.8%) find knowledge about rare diseases with oromaxillofacial manifestation to be a crucial element in the differential diagnosis process as part of the daily routine of dental practice.

## 4. Discussion

To the best of our knowledge, the current study is the first to assess levels of knowledge and associated factors among Israeli dental practitioners regarding rare diseases with orofacial manifestations. The study model is based upon the one proposed by Kühne et al. [7], who based theirs on a study by Vandeborne et al. [13]. A total of 309 Israeli dentists participated in the current study.

Most of the dental specialists defined a “rare disease” as “a disease of which no more than 5 of 10,000 people in the EU are affected”, which is the correct answer [6] (see Table 3). The second most common answer was “*A disease of which no more than 5 of 250,000 people in the EU are affected*”. These results are supported by others [7]. Fewer dentists defined a rare disease as “*a life-threatening chronic disease, which is often genetically determined and difficult to cure*”, reflecting the definition used in recent studies [18,19,20]. Most of the dentists responded that the percentage of rare diseases with orofacial manifestations was 5% instead of 15%, the correct answer [5,7] (Table 3). These results may be explained by 45.5–55.9% of the non-OME dentists having never diagnosed a patient with a rare disease (Table 5). However, 70.1–95.2% of the participants had previously treated such a patient, raising the possibility that diagnosis and treatment are independent variables when it comes to knowledge of the prevalence of rare diseases. As more rare diseases are being more recognized [18], further education of dentists in this field is needed. 

Most dentists estimated that the time lapse until the establishment of a firm diagnosis of a rare disease with orofacial manifestations is between 6 and 18 months. The correct answer is that it takes longer than 3 years, about 4.8 years on average [9,10]. These results emphasize the need to promote ways to enhance our understanding of rare diseases in order to expedite their diagnoses [18], perhaps utilizing our “gut feeling” in such cases [8], and utilizing innovative methods [10].

An outcome variable termed “Total rare diseases index” was formulated and used as a dependent variable in a linear regression analysis (Table 4). Among the various independent variables, being younger, dental education and certification in Israel and working in both private and public settings were significantly associated with the variable of being familiar with higher numbers of rare diseases with orofacial manifestation. We assume that most dental students graduate at a young age; therefore, it may be that the acquired knowledge had not yet been forgotten among the young study participants. Rare diseases are usually taught in oral medicine, oral pathology, and oral surgery courses. In the current study, most dentists graduated in Israel, followed by those who graduated in Eastern Europe, the former USSR and Russia. In Israel, the information on rare diseases is delivered via lectures and derived from compulsory reading of US-based literature [21], which may be different from the curricula of the EU and Russian dental schools. Dental work settings may also be contributory: by working in both public and private clinics, the dental clinician is exposed to a wider range of patient characteristics.

Previous experience in diagnosing a patient with a rare disease with orofacial manifestations (β = 0.107, *p* < 0.058) was correlated with higher scores for “Total rare diseases index”. That finding may further strengthen the notion that previous experience in diagnosing those conditions contributes to the general knowledge about rare diseases with orofacial manifestation. Most of our study OMFs had previously diagnosed such patients, unlike the findings of Kühne et al. [7], who reported that more GDPs had diagnosed such patients compared to dental specialists in a specific region in Germany. The differences between their study and ours may lie in the differences in study groups.

The allocation of sufficient numbers of hours to rare diseases education was also significantly associated with a higher score for the “Total rare diseases” index. Such findings may be affected by country of graduation, as previously stated, or the combination of country and time invested. In addition, knowledge about where to receive information about rare diseases also seems to contribute to knowledge about rare diseases with orofacial manifestation. It may be that knowledge about sources of information is also acquired when sufficient knowledge is imparted during dental education. Accordingly, knowledge about rare diseases may be improved by combining those factors. These findings are not surprising, as more academics support the idea of further education about rare diseases [22,23,24], and increasing numbers of reviews about rare diseases [11,25], including those with orofacial manifestations [1,18,26,27,28], are being published.

Table 6 displays the distribution of the various rare diseases with orofacial manifestations included in the current survey according to the participants’ dental specialty. The six most identified conditions were: Down syndrome, ectodermal dysplasia, Bechet’s syndrome, osteogenesis imperfecta, pemphigus vulgaris, and scleroderma. The highest percentages were recorded for the OMFs, which may be explained by oral medicine specialists and oral and maxillofacial surgeons having more extended curricula on oral pathology. Some of these diseases were described in recent reviews about oral manifestations of rare diseases, such as the one by Luo et al. [18]. Pemphigus vulgaris is not so rare in Israel, estimated at 1.6 per 100,000 [29], which may explain its high level of recognition among the study participants. In the study by Kühne et al. [7] conducted in Germany, the six most well-known rare diseases were: Ehlers–Danlos, ectodermal dysplasia, epidermolysis bullosa, Gorlin–Goltz syndrome, Bechet’s syndrome, and X-linked hypophosphatemia. Those authors also observed differences in the numbers of general dentists, dental specialists and dentists working in a university setting. We decided not to group participants based upon a university work setting since there are only two dental schools in Israel. Interestingly, ectodermal dysplasia and Bechet’s syndrome were recognized by the majority of both German and Israeli dentists who participated in the studies.

Healthcare professionals worldwide, including physicians and dentists, participate in various types of continuing medical education (CME), where the majority of such events are usually comprised of one to several days of conferences, lectures and/or workshops. The majority of our dental practitioners (33.6–61.9%) claimed that they require further information on rare diseases with orofacial manifestation. Unlike the OMF group, the GDPs reported that although they require such information, they lack the time to acquire it (39.3% GDP vs. 4.8%, *p* < 0.05, Table 7). Given that GDPs constitute the majority of dental professionals worldwide, including in the current study, it may be that CME must be of the continuous type, e.g., long-term programs offered by universities and/or professional associations, in order to provide more efficient information about rare diseases with orofacial manifestations [30]. In the current study, the Israeli Oral Medicine Association emerged as being the most useful professional resource for information about rare diseases with orofacial manifestations (56.8–75.8%). Along with possible future incorporation of teledentistry [31], collaborations between dental practitioners and others, e.g., big data analysts, are required to exploit ways to provide progress in rare disease knowledge and education [32]. GDPs (60.2%) claimed that most of their knowledge about rare diseases stems from their dental certification studies, compared to the OMF and REST groups (*p* < 0.001) and the PROS group (*p* < 0.01) (Table 7). The dental specialists of all groups claimed that their knowledge was acquired during specialization programs. Interestingly, there were significant differences in the sources of knowledge acquisition, with most prosthodontics claiming that their knowledge about rare diseases with orofacial manifestations is derived from journals (69.7%). There was agreement among all groups that journals serve as important resources of knowledge [30]. Although there is growing interest in social media groups, including dental ones, we were surprised to observe that their relevance to the expansion of knowledge about rare diseases was not as common (4.8–27% of participants) as in other milieus. This may indicate that dentists wish to acquire their knowledge from recognized and well-respected institutions and associations.

Physicians and dental practitioners may encounter patients with rare diseases as part of their everyday practice, since those diseases may be more common than expected. Elliot and Zuryinski [33] reported that about 8% of the Australian general population live with a rare disease. Nonetheless, many practitioners feel that they do not have enough knowledge and information about such topics [7,9,13]. In a recent systematic review, specific barriers to promote different studies about such topics included the rarity of such diseases, scattering of patients, limited knowledge about disease course, and difficulties to achieve diagnosis of rare diseases (as evidenced by the long period of time to diagnosis) [12]. In the current study, most of the dental practitioners indicated that they require more information about different treatment modalities for rare diseases (Table 7). As supported by Croft et al. [34], it may be that most dentists think in a more “patient prognosis” manner rather than focus upon establishing a diagnosis. Such ways of clinical thinking may not be limited solely to rare diseases with orofacial manifestations. Thinking in a “patient prognosis” manner entails using a wide array of factors and sources of information to guide further clinical decisions, including the choice of treatment modalities. Working under such a mindset allows for the incorporation of various factors, such as genetic and social, to be a part of rare disease case management. 

Genetic factors seem to have and will continue to have important roles in the field of rare diseases; for example, next-generation sequencing [35] to aid in further diagnostic and research about such fields. Social factors, such as patient support groups for those diagnosed with a rare disease as well as for their family members, may play an important role among the different treatment modalities [36]. As evidenced by patients with both rare and chronic diseases, the importance of listening to their symptoms may aid in alleviating them [37,38], e.g., aiding in oro-motor dysfunction in patients with rare diseases with orofacial manifestation [28], and in improving their oral hygiene [39]. In addition, innovations in dental fields, e.g., via the utilization of artificial intelligence of different factors, such as genetic, proteomic and metabolic, may further expand capabilities of diagnosis, prognosis, and treatment of those living with a rare disease with orofacial manifestation [40]. 

The current study has some limitations. Its cross-sectional design restricts the interpretation of results and their extrapolation to other issues. The survey included questions about age of each participant and his/her years of experience. The latter was omitted from the regression analysis because collinearity issues were found for such factors during the regression analysis. The number of dental specialists who participated in the current study may be moderate, but this may be due to their limited numbers in Israel [17]. We do believe, however, that the results of the current study shed light upon the factors that influence the levels of knowledge of rare diseases with orofacial manifestations among dental practitioners. 

In sum, rare diseases with orofacial manifestations are important diagnostic entities, as agreed upon by the majority of participants in the current survey (66.7–81.8%, Table 7), thereby emphasizing the importance of further arming dental care providers with more knowledge about such an important element of patient care.

## 5. Conclusions

Overall, the current study provides insights about levels of knowledge and associated factors of rare diseases with orofacial manifestations among Israeli dentists. As more evidence on novel diagnostic approaches and treatment modalities for such rare diseases is continuously accumulated and reported, this topic gains more attention from both medical and dental audiences. As seen worldwide, such “rare diseases” may be more common than generally believed yet diagnosed late. Factors such as age, country of dental studies and certification, dental clinic work setting, and knowledge acquired and obtained during dental studies and dental specialization programs may enhance our understanding of rare diseases with orofacial manifestation and expedite diagnosis. The current survey revealed that the majority of dental practitioners in Israel claim that they require further information about these rare pathologies. We therefore recommend that tailored CME programs be recruited to answer to these needs.

## Figures and Tables

**Table 1:** Basic demographics of entire dental sample (*n* = 309).

Age Groups (*N*, % of Participants)	Sex (*N*, % of Participants)	Years of Experience (*N*, % of Participants)	Dental Specialty (*N*, % of Participants)	Country of Graduation (*N*, % of Participants)	Work Setting (*N*, % of Participants)
Under 30 y: 16, 5.2% 31–40 y: 80, 25.9% 41–50 y: 83, 26.9% 51–60 y: 69, 22.3% Above 60 y: 61, 19.7%	Male: 192, 62.1% Female: 117, 37.9%	Below 5 y: 39, 12.6% 5–10 y: 42, 13.6% 11–15 y: 35, 11.3% 16–20 y: 40, 12.9% Over 20 y: 153, 49.5%	GP: 211, 68.3% Oral Medicine: 8, 2.6% Orthodontics: 10, 3.2% Oral & maxillofacial surgery: 13, 4.2% Periodontics: 15, 4.9% Endodontics: 10, 3.2% Oral rehabilitation: 33, 10.7% Pedodontics: 9, 2.9%	Israel: 225, 72.8% Eastern Europe (excl. USSR/Russia): 44, 14.2% Former USSR: 10, 3.2% Russia: 5, 1.6% Western Europe (incl. Turkey): 9, 2.9% North America (USA, Canada): 2, 0.6% Middle America: 3, 1% South America: 6, 1.9% Africa: 1, 0.3% Asia (Inc. Jordan, Egypt): 4, 1.3% Oceania (Australia, New-Zealand): 0	Public: 43, 13.9% Private: 152, 49.2% Public and Private: 109, 35.3% Military: 5, 1.6%

**Table 2:** Basic demographics of combined data (*n* = 309).

Country of Graduation (*N*, % of Participants)	Dental Specialty (*N*, % of Participants)
Israel: 225, 72.8% Rest of world: 84, 27.2%	General dental practitioners (GDP): 211, 68.3% Specialists in oral medicine or oral & maxillofacial surgery (OMF): 21, 6.8% Specialists in Orthodontics, Periodontics, Pedodontics, Endodontics (REST): 44, 14.2% Specialists in Prosthodontics (PROS): 33, 10.7%

**Table 3:** Breakdown of participants answering questions regarding definitions, prevalence (3a,b) and diagnosis (3c) of rare diseases.

*In your opinion, a rare disease is?*															
	I do not know how to define a rare disease				A life-threatening chronic disease, which is often genetically determined and difficult to cure					A disease of which no more than 5 of 10,000 people in the EU are affected (CORRECT ANSWER)			A disease of which no more than 5 of 250,000 people in the EU are affected		
General dental practitioners (N, % within group)	27 (12.8%)				19 (9%)					92 (43.6%)			73 (34.6%)		
Specialists in oral medicine or oral & maxillofacial surgery (N, % within group)	2 (9.5%)				1 (4.8%)					12 (57.1%)			6 (28.6%)		
Specialists in Orthodontics, Periodontics, Pedodontics, or Endodontics (N, % within group)	2 (4.5%)				3 (6.8%)					20 (45.5%)			19 (43.2%)		
Specialists in Prosthodontics (N, % within group)	8 (24.2%)				4 (12.1%)					12 (36.4%)			9 (27.3%)		
***How many percent of rare diseases manifest themselves in the craniomaxillofacial region?***															
			5%			7.5%			12%			15% (CORRECT ANSWER)			32%
General dental practitioners (N, % within group)			92 (43.6%)			48 (22.7%)			41 (19.4%)			21 (10%)			9 (4.3%)
Specialists in oral medicine or oral & maxillofacial surgery (N, % within group)			7 (33.3%)			4 (19%)			2 (9.5%)			4 (19%)			4 (19%)
Specialists in Orthodontics, Periodontics, Pedodontics, or Endodontics (N, % within group)			15 (34.1%)			11 (25%)			7 (15.9%)			6 (13.6%)			5 (11.4%)
Specialists in Prosthodontics (N, % within group)			12 (36.4%)			5 (15.2%)			10 (30.3%)			6 (18.2%)			0
***Please estimate, how long does it take for a rare disease with orofacial manifestation to be diagnosed as such after the first appearance of symptoms?***															
		Within 1^st^ month		Within 1–6 months			Within 6–18 months	Within 1.5–3 years			After more than 3 years (CORRECT ANSWER)			No estimation	
General dental practitioners (N, % within group)		11 (5.2%)		54 (25.6%)			56 (26.5%)	30 (14.2%)			7 (3.3%)			53 (25.1%)	
Specialists in oral medicine or oral & maxillofacial surgery (N, % within group)		2 (9.5%)		7 (33.3%)			5 (23.8%)	2 (9.5%)			1 (4.8%)			4 (19%)	
Specialists in Orthodontics, Periodontics, Pedodontics, or Endodontics (N, % within group)		0		6 (13.6%)			14 (31.8%)	10 (22.7%)			3 (6.8%)			11 (25%)	
Specialists in Prosthodontics (N, % within group)		1 (3%)		7 (21.2%)			13 (39.4%)	1 (3%)			3 (9.1%)			8 (24.2%)	

**Table 4:** Linear regression analysis utilizing the outcome variable “*Total rare diseases index*” as the dependent variable.

	Unstandardized Coefficients		Standardized Coefficients	t	
	B	Std. Error	Beta		Sig.
Age	−0.698	0.139	−0.253	−5.015	<0.001 **
Sex	0.595	0.348	0.088	1.711	0.088
Country of Graduation (Israel, Rest of world)	−2.645	0.372	−0.358	−7.103	<0.001 **
Work setting	0.316	0.146	0.107	2.165	0.031 *
How do you asses your knowledge regarding rare diseases?	0.108	0.132	0.049	0.824	0.410
Do you think your knowledge regarding rare diseases is sufficient?	0.307	0.207	0.091	1.482	0.139
Have you ever treated a rare diseased patient?	−0.207	0.410	−0.027	−0.504	0.614
While checking, do you think about rare dis as part of differential diagnosis?	0.062	0.380	0.008	0.162	0.871
Had you ever diagnosed a rare disease with oro-facial manifestation?	0.410	0.215	0.107	1.905	0.058
During your studies, were enough hours allocated about rare diseases?	0.334	0.149	0.111	2.244	0.026 *
Did you participate in courses with special focus on rare diseases?	0.129	0.252	0.026	0.512	0.609
Do you know where to receive info about rare diseases?	0.901	0.410	0.117	2.199	0.029 *

* *p* < 0.05; ** *p* < 0.001.

**Table 5:** Breakdown of dentists with previous diagnosis or treatment of a patient having a rare disease with oromaxillofacial presentation.

*Have you ever treated a patient affected by a rare disease/have you ever seen such a patient before?*			
	No	Yes	
General dental practitioners (N, % within group)	63 (29.9%)	148 (70.1%)	
Specialists in oral medicine or oral & maxillofacial surgery (N, % within group)	1 (4.8%)	20 (95.2%)	
Specialists in Orthodontics, Periodontics, Pedodontics, or Endodontics (N, % within group)	5 (11.4%)	39 (88.6%)	
Specialists in Prosthodontics (N, % within group)	4 (12.1%)	29 (87.9%	
***Have you ever diagnosed a rare disease with oro-facial manifestation?***			
	No	Yes, once	Yes, more than once
General dental practitioners (N, % within group)	118 (55.9%)	51 (24.2%)	42 (19.9%)
Specialists in oral medicine or oral & maxillofacial surgery (N, % within group)	3 (14.3%)	0	18 (85.7%)
Specialists in Orthodontics, Periodontics, Pedodontics, or Endodontics (N, % within group)	21 (47.7%)	11 (25%)	12 (27.3%)
Specialists in Prosthodontics (N, % within group)	15 (45.5%)	4 (12.1%)	14 (42.4%)

**Table 6:** Breakdown of recognition of the different 14 rare diseases with possible manifestation in the oromaxillofacial region.

	General Dental Practitioners (N, % within Group)	Specialists in Oral Medicine or Oral & Maxillofacial Surgery (*N*, % within Group)	Specialists in Orthodontics, Periodontics, Pedodontics, or Endodontics (*N*, % within Group)	Specialists in Prosthodontics (*N*, % within Group)
Down syndrome	164 (77.5%)	19 (90.5%)	37 (84.1%)	27 (81.8%)
Ehlers–Danlos syndrome	69 (32.7%)	18 (85.7)	26 (59.1%)	10 (30.3%)
Ectodermal dysplasia	164 (77.8%)	21 (100%)	39 (88.6%)	32 (97%)
Epidermolysis bullosa	102 (48.3%)	20 (95.2%)	27 (61.4%)	20 (60.6%)
Fetal alcohol syndrome	62 (29.4%)	10 (47.6%)	25 (56.8%)	7 (21.2%)
Gorlin–Goltz syndrome	68 (32.2%)	19 (90.5%)	22 (50%)	7 (21.2%)
Behçet’s disease	170 (80.6%)	21 (100%)	36 (81.8%)	26 (78.8%)
Crohn’s disease	119 (56.4%)	17 (81%)	30 (68.2%)	18 (54.5%)
Osteogenesis imperfecta	174 (82.5%)	21 (100%)	36 (81.8%)	30 (90.9%)
Bullous pemphigoid	128 (60.7%)	20 (95.2%)	34 (77.3%)	22 (66.7%)
Pemphigus vulgaris	184 (87.2%)	20 (95.2%)	37 (84.1%)	29 (87.9%)
Scleroderma	139 (65.9%)	20 (95.2%)	31 (70.5%)	31 (93.9%)
Von Willebrand–Jürgens syndrome	119 (56.4%)	16 (76.2%)	21 (47.7%)	20 (60.6%)
X-linked-Hypophosphataemia	67 (31.8%)	12 (57.1%)	22 (50%)	10 (30.3%)

**Table 7:** Tables providing distribution of answers related to sources of knowledge about rare diseases domain. Some depicts post-hoc Scheffe comparisons, while the rest do not contain such comparisons as the results were not different in a statistically significant manner.

*Do you need information concerning rare diseases with orofacial manifestations in your everyday dental practice*																							
			No, Because I am not Interested in Such Information				No, I am Sufficiently Informed					Yes, but Unfortunately, I Do not Have Time for Research					Yes, but I Do not Know where to Get This Information					Yes	
General dental practitioners (N, % within group)			12 (5.7%)				25 (11.8%)					83 (39.3%) *					32 (15.2%)					71 (33.6%)	
Specialists in oral medicine or oral & maxillofacial surgery (N, % within group)			0				6 (28.6%)					1 (4.8%) *					1 (4.8%)					13 (61.9%)	
Specialists in Orthodontics, Periodontics, Pedodontics, or Endodontics (N, % within group)			4 (9.1%)				8 (18.2%)					11 (25%)					5 (11.4%)					18 (40.9%)	
Specialists in Prosthodontics (N, % within group)			2 (6.1%)				4 (12.1%)					12 (36.4%)					3 (9.1%)					14 (42.4%)	
Post hoc Scheffe												1 > 2 *											
***Which of the following sources do you use as source of information about rare diseases?***																							
	**Social media (facebook, youtube, etc.)**				**Dental friends/colleagues**					**Knowledge I acquired during my dental studies (excluding dental specialization, if applicable**					**Knowledge I acquired during my dental studies (including dental specialization, if applicable**					**Journals**			**dedicated dental forums**
General dental practitioners (N, % within group)	57 (27%)				141 (66.8%)					127 (60.2%) *^,^**					31 (14.7%) *^,^**					106 (50.2%)			75 (35.5%)
Specialists in oral medicine or oral & maxillofacial surgery (N, % within group)	1 (4.8%)				10 (47.6%)					1 (4.8%) *					16 (76.2%) *^,^**					20 (95.2%)			11 (52.4%)
Specialists in Orthodontics, Periodontics, Pedodontics, or Endodontics (N, % within group)	9 (20.5%)				28 (63.6%)					12 (27.3%) *					34 (77.3%) *					31 (70.5%)			13 (29.5)
Specialists in Prosthodontics (N, % within group)	7 (21.2%)				25 (75.8%)					10 (30.3%) **					13 (39.4%) *^,^**					23 (69.7%)			8 (24.2%)
Post-hoc Scheffe										1 > 2 *,3 *,4 **					1 < 2 *,3 *,4 ** 2 > 4 *; 3 > 4 **								
***Which of the following organizations, websites and sources of information about rare diseases with orofacial manifestations do you know?***																							
		**Israeli Oral Medicine Association**				**Israeli Dental Association**					**Other Dental Specialty Association in Israel**							**Foreign Dental Associations**				**None of the Above**	
**General dental practitioners (N, % within group)**		160 (75.8%)				64 (30.3%)					63 (29.9%)							40 (19%)				28 (13.3%)	
**Specialists in oral medicine or oral & maxillofacial surgery (N, % within group)**		14 (66.5%)				4 (19%)					11 (52.4%)							8 (38.1%)				2 (9.5%)	
**Specialists in Orthodontics, Periodontics, Pedodontics, or Endodontics (N, % within group)**		25 (56.8%)				12 (27.3%)					17 (38.6%)							13 (29.5%)				9 (20.5%)	
**Specialists in Prosthodontics (N, % within group)**		20 (60.6%)				4 (12.1%)					13 (39.4%)							6 (18.2%)				9 (27.3%)	
***I need information about rare diseases with orofacial manifestations concerning***																							
				*Incidence and prevalence*					*Lethality and mortality*					*Treatment options*					*Relevant medications*				
**General dental practitioners (N, % within group)**				118 (55.9%)					90 (42.7%)					172 (81.5)					146 (69.2%)				
**Specialists in oral medicine or oral & maxillofacial surgery (N, % within group)**				7 (33.3%)					7 (33.3%)					19 (90.5%)					12 (57.1%)				
**Specialists in Orthodontics, Periodontics, Pedodontics, or Endodontics (N, % within group)**				20 (45.5%)					11 (25%)					34 (77.3%)					21 (47.7%)				
**Specialists in Prosthodontics (N, % within group)**				19 (57.6%)					17 (51.5%)					28 (84.8%)					15 (45.5%)				
***Do you, as a dentist, consider it important to have knowledge about rare diseases with orofacial manifestation?***																							
		no, rare diseases virtually play no role at all in everyday dental practice						no, it is not important					one should have heard about rare diseases			yes, knowledge about rare diseases has an important differential diagnostic significance					yes, it is a very important field		
**General dental practitioners (N, % within group)**		6 (2.8%)						1 (0.5%)					66 (31.3%)			144 (68.2%)					65 (30.8%)		
**Specialists in oral medicine or oral & maxillofacial surgery (N, % within group)**		0						1 (4.8%)					5 (23.8%)			14 (66.7%)					6 (28.6%)		
**Specialists in Orthodontics, Periodontics, Pedodontics, or Endodontics (N, % within group)**		1 (2.3%)						0					11 (25%)			36 (81.8%)					10 (22.7%)		
**Specialists in Prosthodontics (N, % within group)**		2 (6.1%)						0					12 (36.4%)			22 (66.7%)					11 (33.3%)		

* post-hoc Scheffe comparison yielded a significant difference, *p* < 0.001; ** post-hoc Scheffe comparison yielded a significant difference, *p* < 0.01.

## Data Availability

Not applicable.

## Supplementary Materials

The following are available online, https://www.mdpi.com/article/10.3390/ijerph18136830/s1. Table S1: Table summarizing coding of reported self-measures.
