# Supplementary material for: Knowledge and Associated Factors about Rare Diseases among Dentists in Israel: A Cross Sectional Survey
Source: Int J Environ Res Public Health. 2021 Jun 25;18(13):6830. doi: 10.3390/ijerph18136830 (PMC8297074; doi:10.3390/ijerph18136830)
Supplement: Supplementary file 1 [file ijerph-18-06830-s001.zip › ijerph-1244628-supplementary.pdf]

Table S1. Table summarizing coding of reported self-measures.

Variable	Coding
Familiarization with rare diseases presenting with craniomaxillofacial expression	
How do you assess your level of knowledge about rare diseases?	'1 = inadequate', '2 = nearly adequate', '3=adequate', '4=nearly good', '5=good', '6=very good', '7=excellent'
In your opinion, a rare disease is…?	Single choice, coded as '1=I don't know to define a rare disease', '2=a life-threatening chronic disease, which is often genetically determined and difficult to cure', '3=a disease of which no more than 5 of 10,000 people in the EU are affected' (CORRECT ANSWER), '4=a disease of which no more than 5 of 250,000 people in the EU are affected'
What percent of rare diseases manifest themselves in the craniomaxillofacial region?	Single choice, coded as '1=5%', '2=7.5%', '3=12%', '4=15%' (CORRECT ANSWER), '5=32%
Please estimate how long it takes for a rare disease with orofacial manifestation to be diagnosed as such after the first appearance of symptoms	Single choice, coded as '1= within the first month', '2=between 1 to 6 months', '3= between 6 to 18 months', '4= between 1.5 to 3 years', '5=after more than 3 years' (CORRECT ANSWER), '6=no estimation'
Do you believe that your knowledge of rare diseases is sufficient?	Single choice, coded as '1=not at all', '2=slightly sufficient', '3=moderately sufficient', '4=very sufficient', '5=extremely sufficient
Experience with rare diseases	
Have you ever treated a patient affected by a rare disease/have you ever seen such a patient before?	'1=no', '2=yes'
While performing a clinical and radiographic examination, do you think about a possible diagnosis of a rare disease as part of the anamnesis procedure?	'1=no', '2=yes'
Have you ever diagnosed a rare disease with orofacial manifestations?	'1=no', '2=yes, once', '3=yes, several times'
Was any time spent on acquiring knowledge about rare diseases with orofacial manifestations, including their diagnosis and therapy, during your dental education?	'1=no', '2=no, that is why I have a lack of knowledge about rare diseases with orofacial manifestations', '3=yes', '4=yes, but too little time was spent on acquiring information about them', '5=yes, sufficient time was spent on acquiring information about them'
Have you attended training courses with focus on rare diseases with orofacial manifestations?	'1= no, I am not interested in training courses with focus on rare diseases', '2=no, but I would like to attend such courses', '3=yes'
Do you know where to get information about diagnostics, course of disease and therapy when treating a patient affected by a rare disease with orofacial manifestations?	'1=no', '2=yes'
Sources of knowledge about rare diseases	
Do you need information on rare diseases with orofacial manifestations in your everyday dental practice?	multiple answers, coded as '1=no, because I am not interested in such information', '2=no, I am sufficiently informed', '3=yes, but, unfortunately, I do not have time for research', '4=yes, but I do not know where to get this information', '5=yes'

Which of the following sources do you use for information about rare diseases?	multiple answers, coded as '1=social media (Facebook, YouTube, etc.)', '2=dental friends/colleagues', '3=knowledge I acquired during my dental studies (excluding dental specialization, if applicable)', '4= knowledge I acquired during my dental studies (including dental specialization, if applicable)', '5=journals', '6=dedicated dental forums'
Which of the following organizations, websites and sources of information about rare diseases with orofacial manifestations do you know?	multiple answers, coded as '1=Israeli oral medicine association', '2=Israeli dental association', '3=other dental specialty association in Israel (pedodontics, periodontics, orthodontics, prosthodontics, oral and maxillofacial surgery)', '4=nin-Israeli dental associations', '5=none of the above'
I need information about rare diseases with orofacial manifestations in terms of…	multiple answers, coded as '1=incidence and prevalence', '2=lethality and mortality' ,'3=treatment options', '4=relevant medications'
Do you, as a dentist, consider it important to have knowledge about rare diseases with orofacial manifestation?	Multiple answers, coded as '1=no, rare diseases play virtually no role in everyday dental practice', 2=no, it is not important', '3= one should have heard about rare diseases', '4=yes, knowledge about rare diseases has an important differential diagnostic significance', '5=yes, it is a very important field'
